# Optimal threshold of portal pressure gradient for patients with ascites after covered TIPS: a multicentre cohort study

**DOI:** 10.1007/s12072-024-10742-x

**Published:** 2024-11-09

**Authors:** Yifu Xia, Jun Tie, Guangchuan Wang, Hao Wu, Yuzheng Zhuge, Xulong Yuan, Guangjun Huang, Zhen Li, Linhao Zhang, Zihao Cai, Chengwei Tang, Chunqing Zhang

**Affiliations:** 1 Department of Gastroenterology, Shandong Provincial Hospital, Shandong University, Jinan, Shandong China; 2 Department of Gastroenterology, Shandong Provincial Hospital Affiliated to Shandong First Medical University, Jinan, Shandong China; 3 National Clinical Research Center for Digestive Diseases and Xijing Hospital of Digestive Diseases, Air Force Medical University, Xi’an, Shaanxi China; 4 Department of Gastroenterology and Hepatology, West China Hospital, Sichuan University, Chengdu, Sichuan China; 5 Department of Gastroenterology, Affiliated Drum Tower Hospital, Nanjing University Medical School, Nanjing, Jiangsu China

**Keywords:** Liver cirrhosis, Portal hypertension, Haemodynamic, Mortality, Clinical outcomes

## Abstract

**Background:**

Transjugular intrahepatic portosystemic shunt (TIPS) is recommended for treating recurrent and refractory ascites. However, determining the target portal pressure gradient (PPG) has been inconclusive. This multicentre cohort study explored the post-TIPS PPG potential range associated with improving survival.

**Methods:**

The study enrolled 276 patients, all of whom underwent covered TIPS for ascites treatment across four medical centers. The cumulative incidences of clinical outcomes were compared among groups categorized by potential PPG thresholds.

**Results:**

During the whole follow-up period with a medium follow-up of 21.6 (7.5, 41.6) months, 122 (44.2%) experienced liver-related death, and 73 (26.4%) patients experienced a recurrence of ascites. Multivariable analysis revealed PPG < 7 mmHg (*p* = 0.007) and the recurrence of ascites (*p* = 0.033) are independent risk factors for survival, while the PPG ≥ 11 mmHg was an independent risk factor for the recurrence of ascites (*p* = 0.012). Patients with ≥ 7 mmHg had a lower rate of liver-related death than patients with post-TIPS PPG < 7 mmHg (51.0% vs 66.6%, *p* = 0.004), while those with post-TIPS PPG ≥ 11 mmHg exhibited a higher cumulative incidence of ascites compared to those with post-TIPS PPG < 11 mmHg (44.6% vs 33.7%, *p* = 0.023). The robustness of the results was confirmed.

**Conclusion:**

Our study highlighted the existence of an optimal post-TIPS PPG range in patients with recurrent and refractory ascites. Patients may experience improved survival and ascites control with a post-TIPS PPG of 7–11 mmHg.

**Graphical Abstract:**

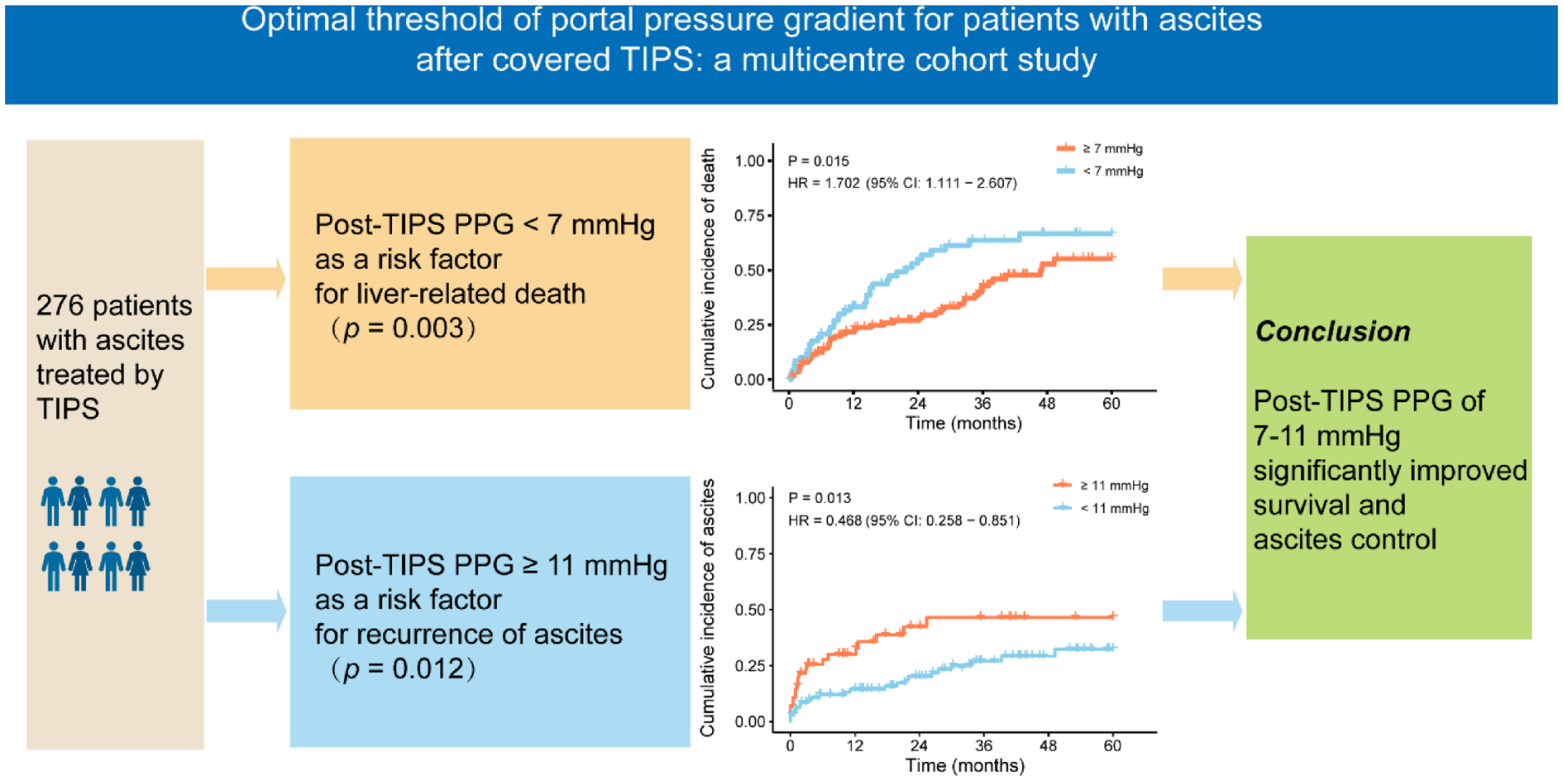

## Introduction

Ascites is the most prevalent decompensation event in patients with cirrhosis, affecting > 50% of such patients and exhibiting an estimated annual incidence of 5–10% in those with compensated cirrhosis [1–3]. The occurrence of ascites results in a 5-year mortality rate of approximately 50–70% [4, 5], and once it becomes refractory, that it cannot be mobilized or whose early recurrence cannot be satisfactorily prevented by medical therapy, median survival is reduced to 6 months [1, 3].

Transjugular intrahepatic portosystemic shunt (TIPS) emerged as an effective treatment for ascites. Notably, studies underscore the superior efficacy of TIPS over paracentesis in controlling recurrent and refractory ascites [6]; and the latest randomised-controlled trial (RCT) concluded that TIPS with covered stents improved transplant-free survival compared with large volume paracentesis (LVP) in patients with recurrent ascites [7]. Current guidelines advocate reducing portal pressure gradient (PPG) to below 12 mmHg or decreased by > 50% after TIPS procedure. However, this standard is primarily used to treat patients with variceal bleeding, there is a lack of consensus on optimal post-TIPS PPG for improving survival, and the optimal PPG decrease to control medically recurrent and refractory ascites needs to be clarified [8]. In addition, the above PPG standard was determined using bare stents, while TIPS with covered stents had better clinical outcomes than that with bare stents; therefore, the standard may not be applicable to covered stents era [9]. The exploration for the appropriate PPG in patients with ascites in only a few studies showing a relationship between post-TIPS PPG and ascites clinical response [10, 11]. Therefore, the determination of the optimal post-TIPS PPG cut-off in patients undergoing covered stent treatment for ascites necessitates further confirmation [1, 8].

We designed this national multicentre cohort study aiming to identify the potential thresholds for post-TIPS PPG that could benefit patients in terms of survival and ascites control.

## Patients and methods

### Patients

This retrospective study included patients who underwent TIPS for recurrent or refractory ascites at four centers between January 2015 and December 2022. All patients received etiological treatment with continued antiviral therapy, alcohol abstinence, or ursodeoxycholic acid depending on the cause of cirrhosis, and receive regular diuretics (spironolactone and furosemide) for ascites, with additional therapeutic paracentesis as needed. Each center had a gastroenterology and hepatology unit with experienced clinicians performing TIPS procedures. The TIPS procedure was carried out using a standard method as previously described under local anesthesia [12]. Three different diameters of covered stents (6, 8, and 10 mm) were used, the selection of stent diameter is mainly based on the operator's experience and patient’s characteristics of pre-TIPS PPG, liver function, and the availability of stents. PPG was obtained preoperatively (pre-TIPS PPG) and immediately after stent placement (post-TIPS PPG). PPG was measured as the difference between the pressure measured in the portal vein and inferior vena cava.

Inclusion criteria were as follows: (1) a diagnosis of cirrhosis (based on clinical signs, laboratory and imaging tests, or liver biopsy) and (2) TIPS procedure for ascites. The exclusion criteria were as follows: (1) age < 14 or > 75 years; (2) not recurrent or refractory ascites; (3) Child–Pugh score > 13; (4) TIPS with uncovered stents, unrelieved bile duct obstruction, hepatocellular carcinoma, or another advanced tumor; (5) no treatment with adequate doses of diuretics; (6) previous TIPS implantation; (7) portocaval surgery or splenectomy; and (8) recurrence of HE without an identifiable trigger (Supplementary Fig. 1).

Recurrent ascites were defined as ≥ 3 LVP within 1 year [8], and refractory ascites were defined per the Asia–Pacific Association for the Study of the Liver [13] as ‘ascites that cannot be mobilized or whose early recurrence cannot be satisfactorily prevented by medical therapy’. All patients received diuretic treatment (at least 100 mg of spironolactone and 40 mg of furosemide daily) [13], tailored to patient tolerance. The study was conducted following the Declaration of Helsinki, approved by the Biomedical Research Ethics Committee of Shandong Provincial Hospital.

### Follow-up

The primary endpoint of this study was liver-related death. Secondary endpoints included the recurrence of ascites, hemorrhage, development of overt hepatic encephalopathy (OHE), and shunt dysfunction. Liver-related death in our study included those due to liver failure, variceal bleeding, and other complications of cirrhosis. Recurrence of ascites was defined as the exacerbation of ascites which could not be improved by diuretics after TIPS, the reappearance of ascites after TIPS, or the requirement of LVP after TIPS. Reduction of ascites was defined as the presence of ascites which was less than that before TIPS with or without diuretics and did not require paracentesis [14]. OHE was defined as hepatic encephalopathy grades ≥ 2 following the West Haven-modified criteria [15, 16]. Shunt dysfunction was suspected at Doppler ultrasonography or CT if the stent was blocked or flow velocity was less than 60 cm/sec or if absence of blood flow or reversal of intrahepatic portal flow occurred [17]. The follow-up period was defined as the duration from admission to death, liver transplantation, the final visit, or study termination. Comprehensive physical examinations, biochemical and hematologic tests, and abdominal ultrasound were conducted.

### Statistical analyses

Quantitative variables were presented as medians (quartiles) and compared using the Mann–Whitney U test for non-normal distribution. Qualitative variables were reported as frequencies (percentages) and compared using the Chi-square test. The density plot and receiver-operating characteristic (ROC) curve were utilized to investigate potential post-TIPS PPG thresholds. Characteristics with *p* value < 0.05 in univariable Cox regression analysis were included in multivariable analysis, and hazard ratios (HRs) with 95% confidence intervals (95% CIs) were reported. Interactive analysis was performed, an interactive *p* value > 0.05 indicates no interaction. The cumulative incidences of outcomes were compared using the Kaplan–Meier method and log-rank test. The competing risk analysis (Fine–Gray test) were also performed, and death or liver transplantation was considered as competing risks for other outcomes, while liver transplantation and non-liver death were considered as competing risks for liver-related death.

A propensity score matching (PSM) was performed on the subgroups with different post-TIPS PPG thresholds due to large baseline differences, the nearest-neighbor matching method with a 0.2 caliper was used to construct the control group. The standard mean difference (SMD) < 0.2 indicated a small difference between groups. All analyses were performed using R v.4.1.0 (http://www.R-project.org/) with the *survival*, *cmprsk*, and *MatchIt* packages. All results with a two-sided *p* value of < 0.05 were considered statistically significant.

## Results

### Patient characteristics and clinical outcomes

The study included 276 patients with recurrent or refractory ascites treated with TIPS. The median age of the patients was 57 years, with 173 (62.7%) being male and 103 (37.3%) being female. One hundred forty patients had hepatitis B virus and received continued antiviral therapy. Hepatitis B virus DNA was detected in 49 (35.0%) patients. A total of 246 patients had varices, among whom 155 patients had a history of variceal bleeding. All of these patients received non-selective beta-blockers or endoscopic therapy according to the guideline for portal hypertension [8]. Partial portal vein thrombosis was diagnosed in 107 patients before TIPS placement, and they all received anticoagulation therapy according to the treatment guideline [8]. All patients were administered furosemide (20–120 mg/d) and spironolactone (40–160 mg/d) before TIPS. The median duration of diuretic use was 24.0 (8.0, 48.0) months. Additionally, all patients underwent at least one LVP procedure, and 162 patients underwent more than three LVP procedures within 12 months. During the TIPS procedure, a gastrorenal shunt was found in eight (2.9%) patients and a splenorenal shunt was found in five (1.8%) patients; all of these patients underwent shunt embolisation. The follow-up period was 21.6 (7.5, 41.6) months. The details of patients’ characteristics are shown in Table 1.

**Table 1:** Demographic and baseline of all patients and group with PPG threshold of 7 and 11 mmHg

Characteristics	All patients (*n* = 276)	< 7 mmHg (*n* = 74)	≥ 7 mmHg (*n* = 202)	*p* value	SMD	< 11 mmHg (*n* = 215)	≥ 11 mmHg (*n* = 61)	*p* value	SMD
Sex				0.053	0.280			0.114	0.258
Male (*n* [%])	173 (62.7)	39 (52.7)	134 (66.3)			129 (60.0)	44 (72.1)		
Female (*n* [%])	103 (37.3)	35 (47.3)	68 (33.7)			86 (40.0)	17 (27.9)		
Age (year)	57 (48, 65)	60 (53, 65)	56 (47, 64)	0.035	0.322	58 (49, 65)	54 (47, 62)	0.029	0.321
Cirrhosis etiology-1				0.483	0.280			0.266	0.369
Hepatic B virus (*n* [%])	140 (50.7)	34 (45.9)	106 (52.5)			111 (51.6)	29 (47.5)		
Hepatic C virus (*n* [%])	13 (4.7)	3 (4.1)	10 (5.0)			9 (4.2)	4 (6.6)		
Alcohol (*n* [%])	34 (12.3)	7 (9.5)	27 (13.4)			27 (12.6)	7 (11.5)		
PBC (*n* [%])	29 (10.5)	11 (14.9)	17 (8.4)			26 (12.1)	3 (4.9)		
Others^†^ (*n* [%])	15 (5.4)	6 (8.1)	10 (5.0)			9 (4.2)	6 (9.8)		
Unknown (*n* [%])	45 (16.3)	13 (17.6)	32 (15.8)			33 (15.3)	12 (19.7)		
Cirrhosis etiology-2				0.336	0.149			0.927	0.034
Hepatic virus (*n* [%])	153 (55.4)	37 (50.0)	116 (57.4)			120 (55.8)	33 (54.1)		
Non-hepatic virus (*n* [%])	123 (44.6)	37 (50.0)	86 (42.6)			95 (44.2)	28 (45.9)		
WBC (10^9^/L)	3.3 (2.3, 5.1)	3.9 (2.2, 5.3)	3.3 (2.3, 5.0)	0.307	0.118	3.3 (2.3, 5.0)	3.5 (2.3, 5.3)	0.473	0.053
RBC (10^12^/L)	3.1 (2.6, 3.5)	3.1 (2.6, 3.5)	3.1 (2.7, 3.6)	0.450	0.142	3.1 (2.6, 3.5)	3.1 (2.7, 3.7)	0.486	0.161
Hemoglobin (g/L)	89 (76, 106)	91 (78, 106)	88 (76, 105)	0.686	0.019	90 (77, 106)	86 (76, 104)	0.586	0.054
Platelet (10^9^/L)	69 (43, 102)	76 (46, 118)	66 (42, 98)	0.112	0.064	71 (43, 104)	55 (38, 85)	0.172	0.003
AST (U/L)	31 (23, 41)	34 (24, 52)	30 (23, 39)	0.058	0.209	31 (23, 43)	29 (19, 36)	0.033	0.261
ALT (U/L)	20 (14, 31)	20 (14, 37)	20 (14, 29)	0.970	0.034	20 (14, 32)	20 (13, 24)	0.151	0.149
GGT (U/L)	33 (20, 61)	36 (22, 63)	33 (19, 61)	0.493	0.066	32 (19, 59)	35 (23, 74)	0.210	0.228
ALP (U/L)	97 (73, 131)	104 (80, 131)	94 (69, 131)	0.198	0.033	94 (73, 126)	104 (75, 140)	0.255	0.162
Total bilirubin (μmol/L)	28 (18, 43)	27 (16, 50)	28 (19, 42)	0.690	0.003	28 (17, 43)	29 (22, 41)	0.550	0.083
Albumin (g/L)	32 (29, 36)	33 (30, 36)	32 (29, 36)	0.823	0.029	32 (29, 36)	33 (30, 37)	0.226	0.188
Prothrombin time (s)	16 (14, 17)	15 (14, 17)	16 (14,17)	0.371	0.129	16 (14, 17)	16 (14,18)	0.726	0.002
INR	1.3 (1.2, 1.5)	1.3 (1.1, 1.5)	1.3 (1.2, 1.5)	0.245	0.139	1.3 (1.2, 1.5)	1.4 (1.2, 1.5)	0.568	0.018
Sodium (mmol/L)	137 (134, 140)	137 (133, 140)	137 (134, 139)	0.563	0.083	137 (134, 140)	137 (134, 140)	0.910	0.026
Creatinine (μmol/L)	82 (67, 104)	94 (68, 110)	79 (66, 103)	0.050	0.290	83 (67, 104)	79 (65, 106)	0.565	0.127
Ascites types				0.916	0.037			0.022	0.341
Recurrent ascites (*n* [%])	59 (21.4)	15 (20.3)	44 (21.8)			39 (18.1)	20 (32.8)		
Refractory ascites (*n* [%])	217 (78.6)	59 (79.7)	158 (79.7)			176 (81.9)	41 (67.2)		
History of bleeding				0.904	0.016			0.825	0.053
Present (*n* [%])	155 (56.2)	42 (56.8)	113 (55.9)			122 (56.7)	33 (54.1)		
Absent (*n* [%])	121 (43.8)	32(43.2)	89 (44.1)			93 (43.3)	28 (45.9)		
History of HE				0.558	0.077			0.906	0.061
Present (*n* [%])	15 (5.4)	5 (6.8)	10 (5.0)			11 (5.1)	4 (6.6)		
Absent (*n* [%])	261 (94.6)	69 (93.2)	192 (95.0)			204 (94.9)	57 (93.4)		
Child–Pugh score (points)	8 (8, 9)	8 (8, 10)	8 (8, 9)	0.709	0.018	8 (8, 9)	8 (8, 9)	0.565	0.036
Child–Pugh class				0.865	0.044			0.917	0.040
B (*n* [%])	209 (75.7)	55 (74.3)	154 (76.2)			162 (75.3)	47 (77.0)		
C (*n* [%])	67 (24.3)	19 (25.7)	48 (23.8)			53 (24.7)	14 (23.0)		
MELD score (points)	11 (8, 14)	12 (8, 15)	11 (8, 14)	0.420	0.063	11 (8, 14)	12 (10, 14)	0.479	0.080
MELD-Na score (points)	13 (9, 17)	13 (9, 18)	12 (9, 17)	0.626	0.017	13 (9, 17)	12 (10, 17)	0.845	0.012
Portal vein thrombosis				0.740	0.064			1.000	0.015
Present (*n* [%])	107 (38.8)	27 (36.5)	80 (39.6)			83 (38.6)	24 (39.3)		
Absent (*n* [%])	169 (61.2)	47 (63.5)	122 (60.4)			132 (61.4)	37 (60.7)		
Varices embolism				0.993	0.021			1.000	< 0.001
Performed (*n* [%])	181 (65.6)	48 (64.9)	133 (65.8)			141 (65.6)	40 (65.6)		
Not-performed (*n* [%])	95 (34.4)	26 (35.1)	69 (34.2)			74 (34.4)	21 (34.4)		
Stent diameter				0.758	0.090			0.316	0.194
6–7 mm (*n* [%])	2 (0.7)	1 (1.4)	1 (0.5)			1 (0.5)	1 (1.6)		
8 mm (*n* [%])	263 (95.3)	70 (94.6)	193 (95.5)			207 (96.3)	56 (91.8)		
10 mm (*n* [%])	11 (4.0)	3 (4.1)	8 (4.0)			11 (4.0)	4 (6.6)		
Pre-TIPS PP (mmHg)	30 (27, 35)	30 (28, 32)	30 (27, 35)	0.843	0.218	27 (24, 30)	35 (30, 38)	0.001	1.472
Pre-TIPS IVCP (mmHg)	6 (3, 8)	5 (3, 7)	7 (4, 8)	0.489	0.400	6 (3, 8)	7 (3, 9)	0.661	0.206
Pre-TIPS PPG (mmHg)	23 (19, 27)	21 (18, 25)	24 (20, 28)	0.005	0.377	22 (18, 26)	27 (23, 30)	< 0.001	0.818
Post-TIPS PP (mmHg)	21 (17, 22)	16 (14, 17)	21 (18, 23)	0.025	1.511	17 (15, 18)	23 (21, 26)	< 0.001	2.123
Post-TIPS IVCP (mmHg)	8 (6, 11)	10 (8, 11)	8 (6, 10)	0.428	0.425)	8 (7, 12)	9 (5, 10)	0.419	0.312
Post-TIPS PPG (mmHg)	9 (7, 10)	6 (4, 6)	10 (8, 11)	< 0.001	2.157	8 (6, 9)	13 (12, 15)	< 0.001	2.183
Reduction of PPG (%)	62 (53, 72)	77 (71, 81)	57 (50, 64)	< 0.001	1.810	65 (57, 74)	50 (42, 56)	< 0.001	1.405

^†^Other etiologies include non-alcoholic fatty liver disease, autoimmune cirrhosis, hepatic sinusoidal obstruction syndrome, Schistosomiasis cirrhosis, and congenital liver fibrosis

PPG, portal pressure gradient; SMD, standardized mean difference; PBC, primary biliary cirrhosis; WBC, white blood cell; RBC, red blood cell; AST, aspartate aminotransferase; ALT, alanine aminotransferase; GGT, gamma-glutamyl transferase; ALP, alkaline phosphatase; INR, international normalized ratio; HE, hepatic encephalopathy; MELD, model for end-stage liver disease; TIPS, transjugular intrahepatic portosystemic shunt; PP, portal pressure; IVCP, inferior vena cava pressure

During the follow-up period, a total of 151 (54.7%) patients died, with 122 (44.2%) of them experiencing liver-related death. Out of the total number of patients, 73 (26.4%) experienced a recurrence of ascites that was uncontrollable with diuretics, while only 11 (4.0%) underwent LVP. The median time to the recurrence of ascites was 2.9 (1, 14.7) months. Among the 68 patients with reduced ascites, five resumed the use of diuretics. Only 28 (10.2%) patients experienced hemorrhage. Among the 136 patients who experienced OHE after TIPS placement, 25 (18.4%) required hospitalization more than once, and three of these patients underwent stent reduction. Meanwhile, 27 (10.2%) patients experienced shunt dysfunction. The outcomes are summarized in Table 2.

**Table 2:** Clinical outcomes during the follow-up period

Clinical outcomes	All patients (*n* = 276)	Patients with PPG < 7 mmHg (*n* = 74)	Patients with PPG ≥ 7 mmHg (*n* = 202)	Patients with PPG < 11 mmHg (*n* = 215)	Patients with PPG ≥ 11 mmHg (*n* = 61)
Death (*n* [%])					
Liver-related death	122 (44.2)	41 (55.4)	81 (40.1)	95 (44.2)	27 (44.3)
ACLF	113 (40.9)	37 (50.0)	76 (37.6)	84 (39.1)	29 (47.5)
HCC	9 (3.3)	4 (5.4)	5 (2.5)	9 (4.2)	0 (0)
Others^§^	29 (10.5)	10 (13.5)	19 (9.4)	22 (10.2)	7 (11.5)
Liver transplantation	5 (1.8)	1 (1.4)	4 (2.0)	4 (1.9)	1 (1.6)
Ascites (*n* [%])					
No ascites	135 (48.9)	34 (45.9)	101 (50.0)	108 (50.2)	27 (44.3)
Reduction	68 (24.6)	21 (28.4)	47 (23.3)	56 (26.0)	12 (19.7)
Recurrence	73 (26.4)	19 (25.7)	54 (26.7)	51 (23.7)	22 (36.1)
Haemorrhage (*n* [%])					
Variceal bleeding	6 (2.2)	0 (0.0)	6 (3.0)	5 (2.3)	1 (1.6)
Unknown	22 (8.0)	6 (8.1)	16 (7.9)	18 (8.4)	4 (6.6)
OHE (*n* [%])					
Grade II	75 (27.2)	18 (24.3)	57 (28.2)	59 (27.4)	16 (26.2)
Grade III	32 (11.6)	10 (13.5)	22 (10.9)	27 (12.6)	5 (8.2)
Grade IV	29 (10.5)	8 (10.8)	21 (10.4)	23 (10.7)	6 (9.8)
Shunt dysfunction (*n* [%]) ^†^	27 (10.2)	3 (4.1)	24 (12.5)	20 (9.6)	7 (12.5)

^§^ Including 8 cases of COVID-19, 2 heart attacks, 3 cerebral Hemorrhages, 2 trauma cases, 2 renal failures, and 12 with unspecified causes

^†^ 264 patients with available data, including 208 patients with PPG < 11 mmHg, 56 patients with PPG ≥ 11 mmHg, 72 patients with post-TIPS PPG < 7 mmHg, and 192 patients with PPG ≥ 7 mmHg

OHE, overt hepatic encephalopathy

### Immediate PPG after TIPS procedure

**Fig. 1:**
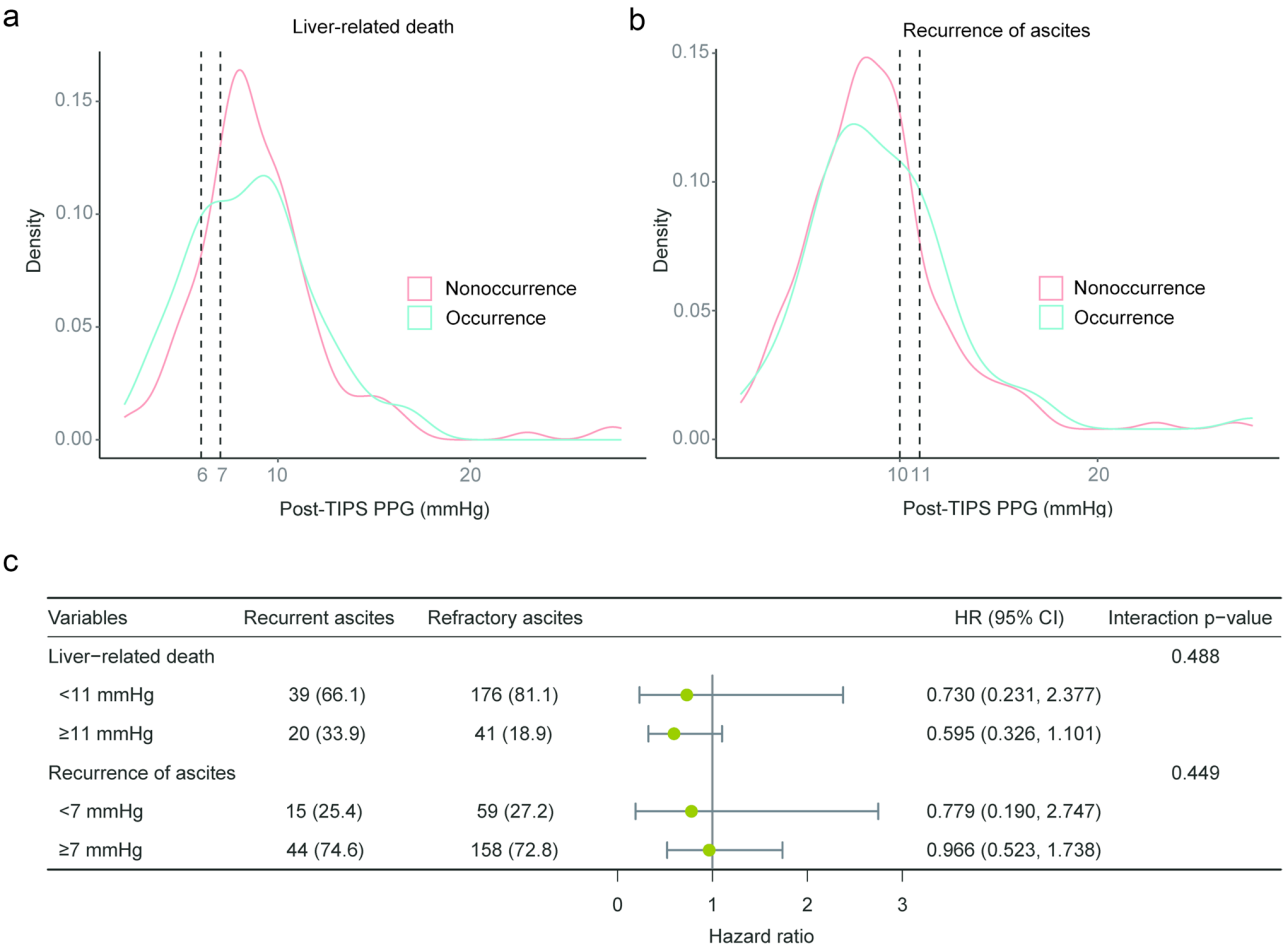
The density plots of post-TIPS PPG and forest plots of interactive analysis. **a, b** Distributions of post-TIPS PPG in patients with ascites and liver-related death are shown with density plots, and (**c**) the results of interactive analysis show no interaction between thresholds of post-TIPS PPG and types of ascites. TIPS, transjugular intrahepatic portosystemic shunt; PPG, portal pressure gradient; CI, confidence intervals; HR, hazard ratio

The PPG was decreased from 23 (19, 27) mmHg to 9 (7, 10) mmHg post-TIPS procedure in all patients, indicating a reduction of 62% (53%, 72%). Based on the information from density plots and the ROC curves, the thresholds for lower mortality may be a post-TIPS PPG of > 7, 8, or 9 mmHg, or a decrease rate of < 75%, and the potential thresholds for ascites control may be < 10 or 11 mmHg for post-TIPS PPG, or a 65% decrease in PPG (Fig. 1A–B). Supplementary Fig. 2–4 displays potential thresholds for other clinical outcomes. The potential thresholds were used as categorical variables for univariable and multivariable regression analyses.

### The effect of PPG reduction on clinical outcomes

The univariable and multivariable analyses of liver-related death revealed that a post-TIPS PPG < 7 mmHg was identified as a risk factor for mortality (*p* = 0.003, HR = 1.803, 95% CI 1.220–2.665) (Supplementary Table 1). Considering other secondary endpoints as influencing factors one by one, we found that the recurrence of ascites (*p* = 0.033, HR = 1.520, 95% CI 1.003–2.235), hemorrhage (*p* = 0.019, HR = 1.911, 95% CI 1.115–3.278), and OHE (*p* = 0.009, HR = 1.666, 95% CI 1.136–2.443) were also risk factors for mortality (Supplementary Table 2). Meanwhile, a post-TIPS PPG < 11 mmHg was a protective factor for ascites control (*p* = 0.012, HR = 0.524, 95% CI 0.316–0.868) (Supplementary Table 3). No specific PPG threshold was identified as an independent factor for other outcomes (Supplementary Table 4–6). The interactive analysis revealed no significant interaction between the PPG < 11 mmHg and recurrent/refractory ascites (*p* = 0.449) or between PPG < 7 mmHg and recurrent/refractory ascites (*p* = 0.488, Fig. 1C).

### The 7 mmHg as a potential PPG threshold for survival

**Fig. 2:**
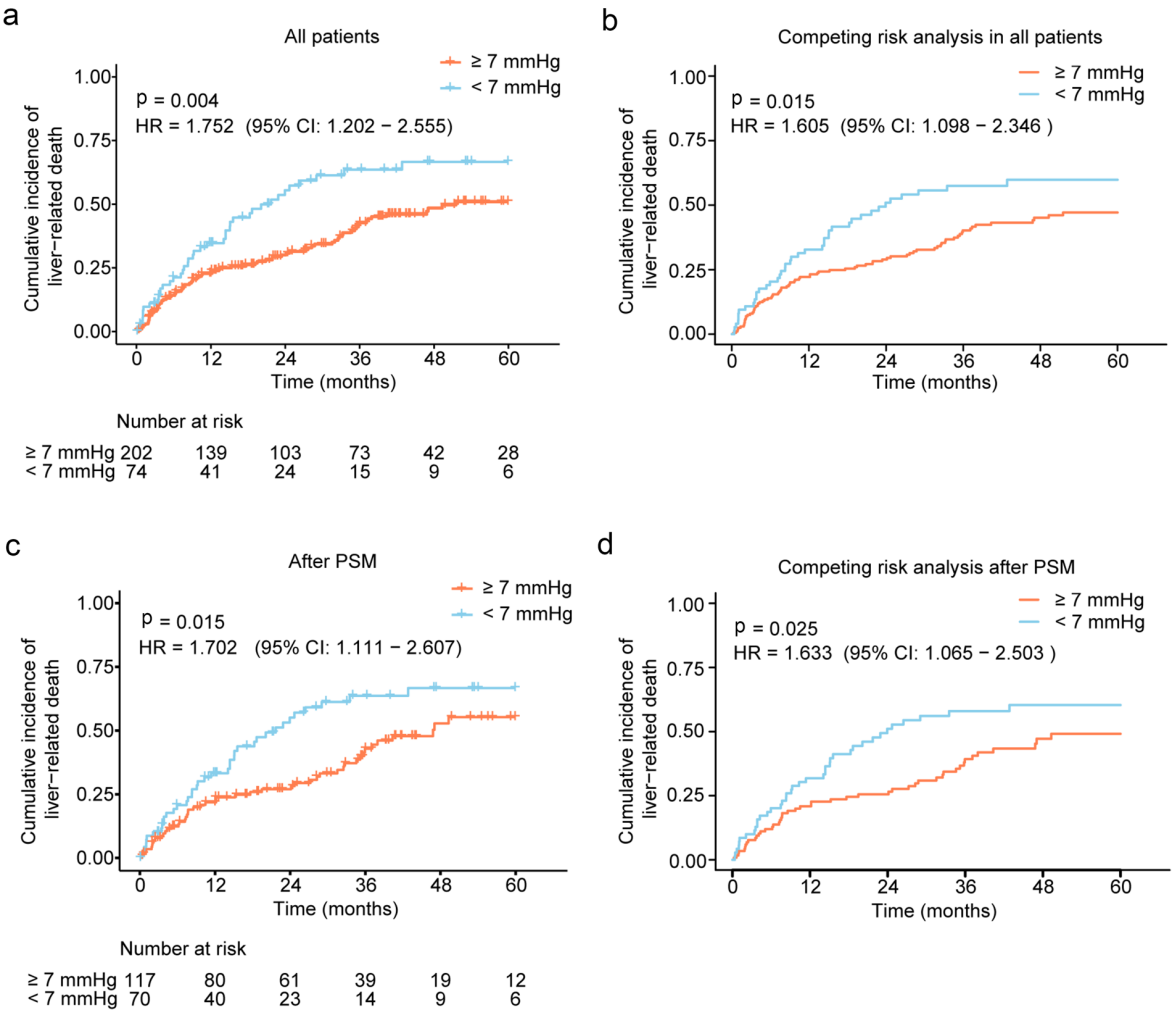
Differences in liver-related mortality between groups with a PPG threshold of 7 mmHg. The differences are showed using Kaplan–Meier curve (**a**) and adjusted with competing risk analysis (**b**) in all patients, which are also performed after PSM (**c–d**). PPG, portal pressure gradient; PSM, propensity score matching; CI, confidence intervals; HR, hazard ratio

According to the post-TIPS PPG of 7 mmHg, 202 patients had post-TIPS PPG ≥ 7 mmHg and 74 had PPG < 7 mmHg (Table 1). Patients with post-TIPS PPG ≥ 7 mmHg had significantly lower mortality than those with PPG < 7 mmHg (51.0% vs 66.6%, *p* = 0.004, HR = 1.752, 95% CI 1.202–2.555), and the difference persisted after accounting for competing events (*p* = 0.015, HR = 1.605, 95% CI 1.098–2.346, Fig. 2A, B). There was no significant difference between the two groups categorized by thresholds of post-PPG < 7 mmHg in recurrence of ascites (35.7% vs 36.3%, *p* = 0.729, HR = 1.097, 95% CI 0.650–1.852), hemorrhage rate (16.5% vs 12.7%, *p* = 0.778, HR = 0.878, 95% CI 0.356–2.169), OHE (55.6% vs 65.7%, *p* = 0.535, HR = 1.128, 95% CI 0.771–1.652), or shunt dysfunction (21.6% vs. 7.5%, *p* = 0.138, HR = 0.403, 95% CI 0.121–1.341) (Supplementary Fig. 5). The Fine–Gray tests demonstrated robust results (Supplementary Fig. 6).

After 2:1 PSM, there were 117 patients in ≥ 7 mmHg group and 70 patients in < 7 mmHg group (Table 3). The significant differences were found in sex, age, aspartate aminotransferase, creatinine, and pre-TIPS PPG between two groups in baseline, and these characteristics were comparable after PSM (Tables 1 and 3, and Supplementary Fig. 7). The survival curve indicated a difference between the two groups in liver-related death (≥ 7 mmHg vs < 7 mmHg, 55.3% vs 66.7%, *p* = 0.015, HR = 1.702, 95% CI 1.111–2.607 Fig. 2C), while no significant difference was observed in other clinical outcomes between two groups (recurrence of ascites: 35.5% vs 37.5%, *p* = 0.647, HR = 1.143, 95% CI 0.645–2.026; hemorrhage, 13.6% vs 13.1%, *p* = 0.771, HR = 1.163, 95% CI 0.422–3.206; OHE, 60.6% vs 64.4%, *p* = 0.941, HR = 1.016, 95% CI 0.664–1.555; shunt dysfunction, 14.8% vs 7.8%, *p* = 0.481, HR = 0.628, 95% CI 0.172–2.288) (Supplementary Fig. 8). The competing risk analysis indicated robust results of significant difference between two groups in liver-related death (*p* = 0.025, HR = 1.633, 95% CI 1.065–2.503 Fig. 2D), and no difference was observed in other clinical outcomes (Supplementary Fig. 9).

**Table 3:** Demographic and baseline of patients with PPG threshold of 7 and 11 mmHg after PSM^§^

Characteristics	< 7 mmHg (*n* = 70)	≥ 7 mmHg (*n* = 117)	*p* value	SMD	< 11 mmHg (*n* = 97)	≥ 11 mmHg (*n* = 56)	*p* value	SMD
Sex			0.583	0.106			1.000	0.006
Male (*n* [%])	37 (52.9)	68 (58.1)			73 (75.3)	42 (75.0)		
Female (*n* [%])	33 (47.1)	49 (41.9)			24 (24.7)	14 (25.0)		
Age (year)	60 (53, 65)	58 (48, 66)	0.663	0.103	54 ± 11.16	53 ± 11	0.748	0.054
Cirrhosis etiology†			0.748	0.071			0.810	0.069
Hepatic virus (*n* [%])	34 (48.6)	61 (52.1)			57 (58.8)	31 (55.4)		
Non-Hepatic virus (*n* [%])	36 (51.4)	56 (47.9)			40 (41.2)	25 (44.6)		
WBC (10^9^/L)	3.7 (2.2, 5.3)	3.3 (2.5, 5.0)	0.879	0.027	3.4 (2.2, 4.8)	3.4 (2.3, 5.2)	0.495	0.140
RBC (10^12^/L)	3.1 (2.6, 3.5)	3.2 (2.8, 3.6)	0.166	0.250	3.0 (2.6, 3.4)	3.0 (2.7, 3.6)	0.633	0.119
Hemoglobin (g/L)	92 (79, 107)	92 (80, 109)	0.713	0.104	88 (71, 105)	85 (76, 97)	0.829	0.070
Platelet (10^9^/L)	76 (46, 117)	71 (45, 103)	0.617	0.058	68 (36, 97)	53 (35, 80)	0.356	0.008
AST (U/L)	34 (24, 51)	30 (24, 42)	0.235	0.121	29 (22, 38)	29 (19, 35)	0.490	0.011
ALT (U/L)	20 (13, 34)	20 (13, 31)	0.941	0.045	20 (14, 31)	20 (13, 24)	0.461	0.025
GGT (U/L)	37 (22, 61)	33 (21, 63)	0.813	0.123	32 (22, 64)	35 (24, 63)	0.608	0.059
ALP (U/L)	104 (80, 131)	97 (70, 136)	0.654	0.095	89 (69, 122)	104 (79, 137)	0.079	0.210
Total bilirubin (μmol/L)	29 (17, 50)	29 (21, 39)	0.972	0.032	27 (17, 42)	30 (22, 41)	0.224	0.059
Albumin (g/L)	33 (30, 37)	31 (29, 36)	0.203	0.128	33 (30, 36)	32 (30, 36)	0.745	0.028
Prothrombin time (s)	15 (14, 17)	16 (14, 17)	0.863	0.067	16 (14, 17)	16 (14, 18)	0.617	0.098
INR	1.3 (1.2, 1.5)	1.3 (1.2, 1.5)	0.523	0.111	1.3 (1.2, 1.5)	1.4 (1.2, 1.5)	0.243	0.231
Sodium (mmol/L)	137 (133, 140)	136 (133, 140)	0.588	0.071	137 (134, 140)	136 (134, 140)	0.486	0.021
Creatinine (μmol/L)	91 (68, 108)	81 (67, 104)	0.314	0.128	81 (67, 104)	79 (65, 94)	0.527	0.202
Ascites types			1.000	0.009			0.061	0.343
Recurrent ascites (*n* [%])	14 (20.0)	23 (19.7)			17 (17.5)	18 (32.1)		
Refractory ascites (*n* [%])	56 (80.0)	94 (80.3)			80 (82.5)	38 (67.9)		
History of bleeding			0.608	0.101			0.258	0.219
Present (*n* [%])	30 (42.9)	56 (47.9)			33 (34.0)	25 (44.6)		
Absent (*n* [%])	40 (57.1)	61 (52.1)			64 (66.0)	31 (55.4)		
History of HE			0.806	0.084			1.000	0.038
Present (*n* [%])	65 (92.9)	111 (94.9)			91 (93.8)	52 (92.9)		
Absent (*n* [%])	5 (7.1)	6 (5.1)			6 (6.2)	4 (7.1)		
Child–Pugh score (points)	9 (8, 10)	8 (8, 9)	0.896	0.005	8 (8, 9)	8 (8, 9)	0.388	0.188
Child–Pugh class			0.752	0.074			0.287	0.211
B (*n* [%])	51 (72.9)	89 (76.1)			81 (83.5)	42 (75.0)		
C (*n* [%])	19 (27.1)	28 (23.9)			16 (16.5)	14 (25.0)		
MELD score (points)	11 (9, 14)	11 (8, 14)	0.587	0.002	11 ± 5	12 ± 4	0.320	0.171
MELD-Na score (points)	13 (10, 17)	13 (9, 17)	0.999	0.025	12 (9, 16)	12 (10, 17)	0.449	0.052
Portal vein thrombosis			0.743	0.073			0.729	0.087
Present (*n* [%])	43 (61.4)	76 (65.0)			53 (54.6)	33 (58.9)		
Absent (*n* [%])	27 (38.6)	41 (35.0)			44 (45.4)	23 (41.1)		
Varices embolism			0.925	0.038			0.953	0.040
Performed (*n* [%])	24 (34.3)	38 (32.5)			33 (34.0)	18 (32.1)		
Not-performed (*n* [%])	46 (65.7)	79 (67.5)			64 (66.0)	38 (67.9)		
Stent diameter			0.346	0.197			0.417	0.191
6–7 mm (*n* [%])	1 (1.4)	0 (0.0)			0 (0.0)	1 (1.8)		
8 mm (*n* [%])	66 (94.3)	114 (97.4)			92 (94.8)	52 (92.9)		
10 mm (*n* [%])	3 (4.3)	3 (2.6)			5 (5.2)	3 (5.4)		
Pre-TIPS PP (mmHg)	30 (28, 32)	30 (26, 35)	0.966	0.081	30 (29, 35)	32 (30, 35)	0.367	0.600
Pre-TIPS IVCP (mmHg)	5 (3, 7)	6 (3, 8)	0.521	0.379	6 (4, 9)	7 (3, 9)	1.000	0.100
Pre-TIPS PPG (mmHg)	22 ± 6	22 ± 5	0.518	0.095	25 (22, 29)	26 (23, 29)	0.407	0.100
Post-TIPS PP (mmHg)	16 (14, 17)	21 (18, 22)	0.036	1.435	17 (15, 18)	22 (21, 24)	0.004	2.122
Post-TIPS IVCP (mmHg)	10 (8, 11)	8 (6, 11)	0.607	0.261	11 (9, 12)	9 (6, 10)	0.344	0.493
Post-TIPS PPG (mmHg)	5 (4, 6)	9 (8, 11)	< 0.001	2.561	8 (7, 10)	12 (12, 14)	< 0.001	2.162
Reduction of PPG [%]	77 (72, 81)	56 (50, 63)	< 0.001	1.977	68 ± 10	48 ± 9	< 0.001	2.089

^§^The groups of 7 mmHg included sex, age, creatinine, and pre-TIPS PPG in PSM process, and the groups of 11 mmHg included sex, age, aspartate aminotransferase, gamma-glutamyl transferase, and pre-TIPS PPG in PSM process

^†^Cirrhosis etiologies were simplified into two categories of viral and non-viral due to the small number of patients associated with other etiologies. Other etiologies include non-alcoholic fatty liver disease, autoimmune cirrhosis, hepatic sinusoidal obstruction syndrome, Schistosomiasis cirrhosis, and congenital liver fibrosis

PPG, portal pressure gradient; PBC, primary biliary cirrhosis; WBC, white blood cell; RBC, red blood cell; AST, aspartate aminotransferase; ALT, alanine aminotransferase; GGT, gamma-glutamyl transferase; ALP, alkaline phosphatase; INR, international normalized ratio; HE, hepatic encephalopathy; MELD, model for end-stage liver disease; TIPS, transjugular intrahepatic portosystemic shunt; PP, portal pressure; IVCP, inferior vena cava pressure

### The 11 mmHg as a potential PPG threshold for ascites control

**Fig. 3:**
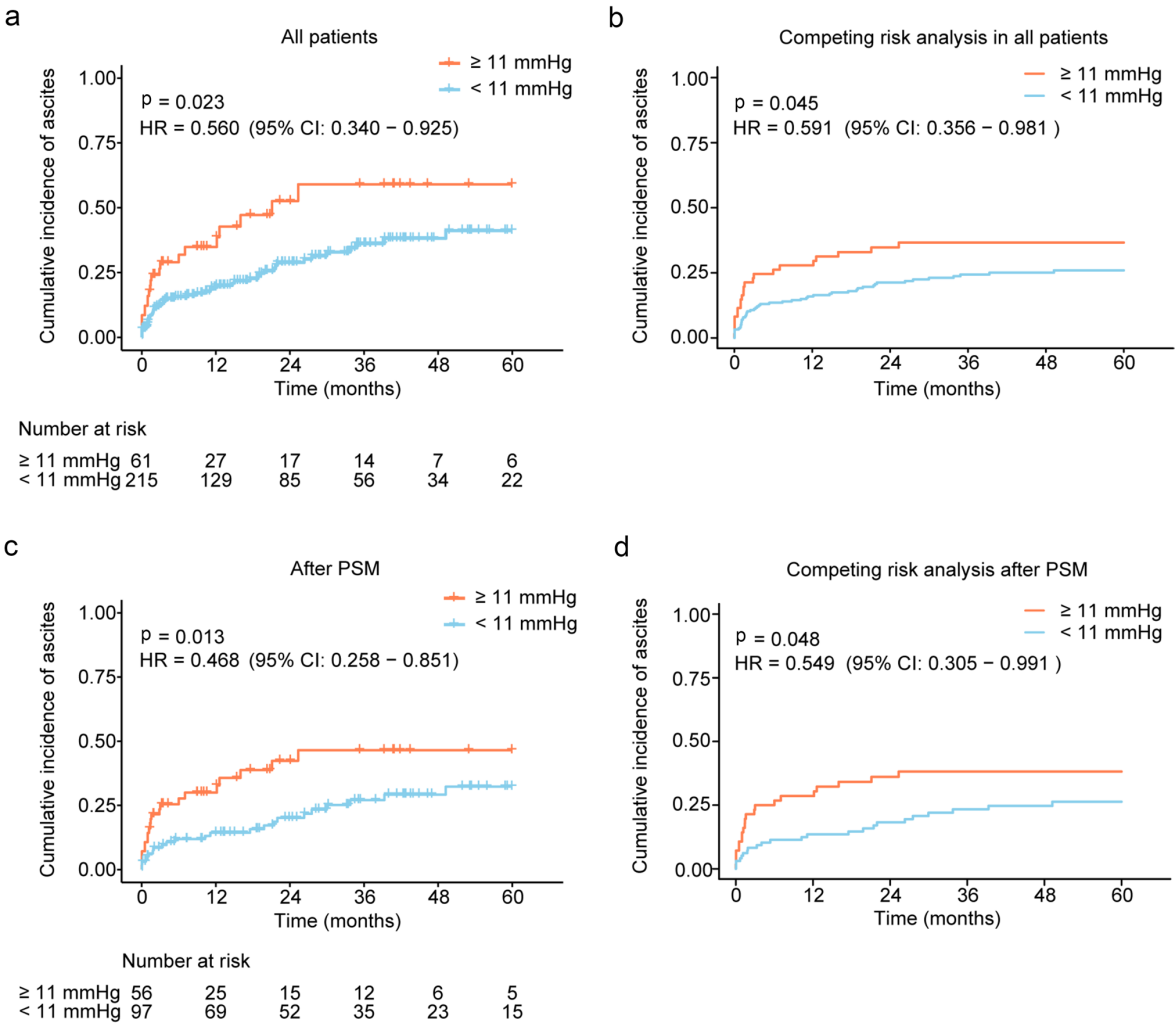
Differences in recurrence of ascites between groups with a PPG threshold of 11 mmHg. The differences are shown using Kaplan–Meier curve (**a**) and adjusted with competing risk analysis (**b**) in all patients, which are also performed after PSM (**c–d**). PPG, portal pressure gradient; PSM, propensity score matching; CI, confidence intervals; HR, hazard ratio

There were 61 patients with post-TIPS PPG ≥ 11 mmHg and 215 with PPG of < 11 mmHg (Table 1). Patients with post-TIPS PPG ≥ 11 mmHg had a significantly higher incidence of recurrence of ascites compared with another group (44.6% vs 33.7%, *p* = 0.023, HR = 0.560, 95% CI 0.340–0.925), and similar patterns were observed after using competing risk analysis (*p* = 0.045, HR = 0.591, 95% CI 0.356–0.981, Fig. 3A, B). There was no significant difference between the two groups categorized by thresholds of post-PPG < 11 mmHg in liver-related death (52.4% vs 56.0%, *p* = 0.974, HR = 0.993, 95% CI 0.648–1.523), hemorrhage rate (13.3% vs 16.5%, *p* = 0.574, HR = 1.320, 95% CI 0.502–3.472), OHE (52.2% vs 58.8%, *p* = 0.266, HR = 1.270, 95% CI 0.833–1.936), or shunt dysfunction (28.8% vs 15.7%, *p* = 0.668, HR = 0.828, 95% CI 0.350–1.959), and the competing risk analysis indicated robust results (Supplementary Fig. 10–11).

After 1:2 PSM, there were 56 patients with post-TIPS PPG ≥ 11 mmHg and 97 with PPG of < 11 mmHg (Table 3). The sex, age, aspartate aminotransferase, gamma-glutamyl transferase, ascites types, and pre-TIPS PPG of patients had significant differences between two groups in baseline, and these characteristics except ascites types were comparable after PSM (Tables 1 and 3, and Supplementary Fig. 12). The survival curve showed a significant statistical difference in ascites incidence post-TIPS (≥ 11 mmHg vs < 11 mmHg, 46.5% vs 32.3%, *p* = 0.013, HR = 0.468, 95% CI 0.258–0.851), and the difference was also observed with competing risk analysis (*p* = 0.048, HR = 0.549, 95% CI 0.305–0.991, Fig. 3C, D). However, patients in groups categorized by 11 mmHg of post-TIPS PPG had a similar cumulative incidences of liver-related death (52.3% vs 52.5%, *p* = 0.602, HR = 0.875, 95% CI 0.529–1.446), hemorrhage (14.2% vs 16.9%, *p* = 0.827, HR = 1.126, 95% CI 0.390–3.245), OHE (55.2% vs 57.4%, *p* = 0.274, HR = 1.302, 95% CI 0.811–2.089), and shunt dysfunction (31.6% vs 15.0%, *p* = 0.355, HR = 0.627, 95% CI 0.233–1.688), and the Fine–Gray test indicated robust results (Supplementary Fig. 13–14).

### Clinical benefit for PPG7-11 patients

We compared the clinical outcomes of three groups of patients based on post-TIPS PPGs of < 7 mmHg, 7–11 mmHg, and ≥ 11 mmHg. The results indicated that patients with a PPG < 7 mmHg had a significantly higher liver-related mortality rate than that of the other two groups (< 7 mmHg vs 7–11 mmHg: 66.6% vs 50.5%, *p* = 0.002, HR = 0.531, 95% CI 0.353–0.798; < 7 mmHg vs ≥ 11 mmHg: 66.6% vs 52.4%, *p* = 0.107, HR = 0.670, 95% CI 0.412–1.091). However, patients with a PPG ≥ 11 mmHg had a higher rate of ascites recurrence than that of the other two groups (< 7 mmHg vs ≥ 11 mmHg: 36.3% vs 44.6%, *p* = 0.242, HR = 1.443, 95% CI 0.781–2.667; 7–11 mmHg vs ≥ 11 mmHg: 32.2% vs 44.6%, *p* = 0.014, HR = 1.983, 95% CI 1.150–3.421). Conversely, patients with PPGs within the range of 7–11 mmHg had lower mortality and ascites recurrence rates (Supplementary Fig. 15 and 16).

The results of the interaction analysis showed no significant interaction between different medical centers and the PPG threshold of < 7 mmHg in terms of survival (*p* = 0.384). Furthermore, no significant interaction between different medical centers and the PPG threshold of ≥ 11 mmHg in terms of ascites control (*p* = 0.319) was observed. Therefore, the results of the thresholds were not influenced by a center effect.

## Discussion

Covered TIPS was recommended for patients with recurrent or refractory ascites [2, 8, 13]; however, the hemodynamic target of post-TIPS PPG was established with the use of bare stents; therefore, the optimal post-TIPS PPG thresholds in patients with ascites need to be examined in covered stents. However, we observed a lack of dedicated studies that specifically examined the relationship between the PPG after covered stent placement and clinical outcomes, and the standard for post-TIPS PPG in patients with ascites is not clear [8]. Logically, greater PPG reduction or lower PPG value after TIPS insertion can achieve more adequate portal pressure reduction, and decrease the risk of ascites recurrence, but are associated with a greater risk of HE. Our study, which highlighted the potential existence of an optimal post-TIPS PPG range for patients with recurrent and refractory ascites, found that patients may experience improved survival and ascites control with a post-TIPS PPG of 7–11 mmHg.

The post-TIPS PPG in our study was 9 [7, 10] mmHg, while it was observed to be 6.4 ± 4.2 mmHg in the study by Bureau et al. [7], which included only patients with covered stents. The post-TIPS PPG was higher in our study, and the potential reason for the difference may be that only 10 mm-diameter stents were used in their study, while most of our patients had 8 mm-diameter stents (95.3%). Although the lower PPG was able to reduce the recurrence of ascites, too low PPG will lead to reduced benefit for patients. Several studies have indicated that an excessively low post-TIPS PPG is a risk factor for survival, with the potential threshold possibly being 5 or 8 mmHg [18–20]. In line with these findings, we observed that patients with post-TIPS PPG < 7 mmHg had significantly higher liver-related mortality than other patients in our study. This threshold may indicate a point at which the risks of aggressive intervention outweigh the benefits of reducing ascites. The impact of low PPG on survival has been previously described and can be attributed to the reduced functional hepatic reserve and increased burden of comorbidities [19, 20]. Meanwhile, the lower PPG generally indicates more shunt of TIPS, which might reduce liver perfusion and lead to impaired liver function in these patients [19, 21]. Therefore, the lower PPG is not always better. The PPG should be controlled in a range that does not increase other complications while reducing the recurrence of ascites, and the lowest PPG threshold in this study was 7 mmHg.

According to the multivariable analysis, the recurrence of ascites was a risk factor of liver-related death in our study. Similarly, the study by Queck et al. observed an appropriate post-TIPS PPG of patients. The difference is that Queck et al. mainly studied patients with bare stents, whereas all of our patients used covered stents. Uncovered stents predictably tend to decrease in diameter over time, as a result, the decrease in PPG achieved post-TIPS is not sustained but progressively lost until reintervention [10]. In contrast, TIPS using covered stents maintains the pressure drop during follow-up [22], supporting a longer observation period; therefore, we were able to obtain clinical outcomes with a longer follow-up period of up to 5 years in our study. Previous studies also have shown that most patients experienced resolution of ascites starting from 6 months after TIPS [13, 23]; therefore, it is more valuable to observe the long-term prognosis of ascites [24]. In addition, our research results also indicated that the recurrence of ascites after TIPS could affect the survival of patients. This suggests that the recurrence of ascites can serve as a predictive indicator for the prognosis of patients after TIPS.

There were only 1.8% patients who underwent liver transplantation in our cohort, the limited availability of liver donors for transplantation, coupled with the exorbitant costs involved, might be the primary reasons behind the low number. The cumulative incidence of OHE was 44.6% within 1 year after TIPS in our study, and the multivariable analysis did not identify post-TIPS PPG as an influencing factor for OHE, which is consistent with findings shown in other studies [25–28]. The use of small-diameter covered stents (8 mm) may have reduced the incidence of OHE after TIPS [9], which may be the reason why the PPG threshold for OHE was not found.

This study has some limitations. First, the retrospective design of our analyses may have introduced some bias. However, we included consecutive patients to minimize excessive bias. Second, the majority of patients in this study received 8-mm stents. Although our results indicated that the stent diameter does not affect the prognosis, further confirmation among populations with different stent diameters is required. Third, we could only collect immediate post-TIPS PPGs retrospectively, which may have varied over time. Further trials are needed to confirm this potential range with long-term PPG after TIPS. Finally, to obtain ample patient data, those with a history of bleeding were not excluded. However, PSM was performed to minimise the influence of bleeding history on the results of the analysis.

In summary, our study provides compelling evidence for the existence of an optimal post-TIPS PPG range for patients with recurrent and refractory ascites. Patients with a post-TIPS PPG of 7–11 mmHg demonstrated significantly improved survival and ascites control. Future prospective, multicentre studies are warranted to refine the recommendations for personalized post-TIPS PPG management.

## Data Availability

The data presented in this article will be made available by the corresponding author upon request.

## Abbreviations

TIPS: Transjugular intrahepatic portosystemic shunt
RCT: Randomised-controlled trial
LVP: Large volume paracentesis
PPG: PORTAL pressure gradient
OHE: Overt hepatic encephalopathy
ROC: Receiver-operating characteristic
HR: Hazard ratio
CI: Confidence intervals
PSM: Propensity score matching
MELD: Model for end-stage liver disease
WBC: White blood cell
RBC: Red blood cell
AST: Aspartate aminotransferase
ALT: Alanine aminotransferase
GGT: Gamma-glutamyl transferase
ALP: Alkaline phosphatase
HE: Hepatic encephalopathy
INR: International normalized ratio
PP: Portal pressure
IVCP: Inferior vena cava pressure
